# Evaluation of a haemozoin-based rapid diagnostic test for diagnosis of imported malaria during the phase of prevention of reestablishment in Sri Lanka

**DOI:** 10.1186/s12936-022-04283-7

**Published:** 2022-09-10

**Authors:** Deepika Fernando, Priyaleela Thota, Saveen Semege, Rahuman Booso, David Bell, Kumudunayana T. de A. W. Gunasekera, Prasad Ranaweera

**Affiliations:** 1 Department of Parasitology, Faculty of Medicine, University of Colombo, Colombo, Sri Lanka; 2 Hemex Health, Portland, OR USA; 3 Army Headquarters, Sri Jayawardenepura, Sri Lanka; 4 Directorate of Health Services, Campaign, Sri Lanka; 5 Anti Malaria Campaign, 555/5 Public Health Building, Narehenpita, Sri Lanka

**Keywords:** Malaria, Diagnosis, Rapid diagnostic test, Haemozoin, Microscopy, Polymerase chain reaction

## Abstract

**Background:**

Sri Lanka, an island nation, has eliminated endemic malaria transmission. Maintaining elimination in the continued presence of vectors requires vigilance in screening people travelling from high malaria-risk areas and a rapid response with focal screening for infections identified in the community. Such screening requires accurate and very rapid assays that enable an immediate response. Both microscopy and rapid diagnostic tests (RDTs) have limitations including sensitivity and speed in screening large numbers, while polymerase chain reaction (PCR) is practical only as laboratory confirmation. This study assessed the utility of ‘Gazelle’, a novel rapid malaria assay based on magneto-optical detection of haemozoin, a by-product of malaria parasite metabolism.

**Methods:**

Between October 2020 and March 2021, two groups of individuals were screened for malaria by four methods, namely, microscopy, Rapid Diagnostic Test (RDT), Gazelle and PCR. Passive case detection was carried out for confirmation of diagnosis amongst individuals suspected of having malaria. Individuals at high-risk of acquiring malaria, namely persons returning from malaria endemic countries, were screened by active case detection.

**Results:**

Of the 440 individuals screened for malaria, nine malaria positives were diagnosed by PCR, microscopy and the HRP2 band of RDT, which included five *Plasmodium falciparum* infections, two *Plasmodium ovale*, and one each of *Plasmodium vivax* and *Plasmodium malariae.* Gazelle correctly detected the *P. vivax, P. ovale* and *P. malariae* infections within the 2 min test time, but did not detect two *P. falciparum* infections giving a sensitivity of 77.8%. Specificity was 100%.

**Discussion:**

The Gazelle, a portable bench top device proved useful to screen a large number of blood samples for non-falciparum parasites within 5 minutes of sample input. Species differentiation, and improvement in *P. falciparum* detection, will be important to broaden utility.

**Supplementary Information:**

The online version contains supplementary material available at 10.1186/s12936-022-04283-7.

## Background

Sri Lanka received malaria-free certification from the World Health Organization (WHO) in September 2016, becoming the second country in the WHO South East Asia region to be declared malaria-free [1]. In the years prior to elimination, frequent epidemics of malaria were recorded, including the massive epidemic of 1934/35. Near elimination state was reached in 1963 when 17 cases of malaria were diagnosed. However, declining financial and political commitment led to resurgence of the disease, which lasted over the next five decades. From 1999, with renewed malaria control policies, the malaria incidence declined until the last case was reported in October 2012 [2]. Prior to elimination, *Plasmodium vivax* infections accounted for over 90% of malaria infections diagnosed. *Plasmodium falciparum* was present but its incidence varied over time and *Plasmodium malariae* was also prevalent till about the late 1960’s after which transmission was interrupted. The Anti Malaria Campaign (AMC) began to classify cases as indigenous and imported since 2008 [2]. Following elimination, with the exception of one introduced and one transfusion induced infection which originated from a case of imported malaria reported in 2018 and 2021, respectively [3, 4], there has been no local transmission of malaria in Sri Lanka.

The number of imported malaria cases reported over 5 years, between 2017–2021, included 97 *P. vivax* cases, 84 *P. falciparum*, 26 *Plasmodium ovale*, 6 *P. malariae,* and one mixed infection. The high number of imported malaria cases being diagnosed combined with high receptivity due to the presence of the primary vector *Anopheles culicifacies* and emergence of *Anopheles stephensi* [5] has led to a risk of reestablishment of the disease in the country. This is further complicated by the presence of a non-immune population, declining physician awareness leading to delayed diagnosis of malaria [6] and dwindling financial support to carry out malaria related surveillance activities.

Two malaria case surveillance strategies play a key role to prevent the reestablishment of malaria in Sri Lanka. Passive Case Detection (PCD), which is the detection of malaria cases amongst people who seek health care on their own, usually for fever; and Active Case Detection (ACD), which involves actively searching for malaria infections, such as in people or populations at high risk but who may or may not be obviously ill [7, 8]. Laboratory confirmation of malaria prior to starting anti-malarial treatment is mandatory in Sri Lanka [9].

Microscopy remains the traditional gold standard for malaria diagnosis in Sri Lanka. Microscopy is performed by Public Health Laboratory Technicians (PHLT), who are posted at government hospitals across the country [2]. Challenges exist in maintaining the proficiency of malaria diagnosis via microscopy in the presence of low case numbers [3, 4]. The Carestart ™ Pf/Pan Combo Rapid Diagnostic Tests (RDTs) which are purchased from WHO-prequalified suppliers are being used as a supplementary diagnostic method by the AMC. RDTs are made available at health care institutions, including those where PHLTs are not available, and medical centres at ports of entry where malaria diagnostic services are available 24 h a day. This test has shown good performance for the detection of symptomatic *P. falciparum* (due to high sensitivity of HRP2) and *P. vivax* but a lower sensitivity has been recorded for the pan specific pLDH for *P. falciparum, P. ovale* and *P. malariae* [10]. This has to be taken into consideration when using this RDT as a point-of-care test whenever microscopy facilities are not available, especially in instances where HRP2 gene deletion is a possibility irrespective of the parasite density [11]. However, there have been challenges where both tests have been negative in patients who subsequently were found to be positive for malaria in Sri Lanka [4, 12]. Such discrepancies in diagnostic test results are confirmed by polymerase chain reaction (PCR), which allows for a more sensitive and specific detection of malaria parasites. Due to the cost of consumables, training and equipment, the use of PCR has been restricted for confirmation of diagnosis when microscopy and RDT give inconclusive results, or to confirm the species of the parasite. A well-developed laboratory infrastructure is available only at the AMC headquarters and it is not cost effective to use molecular diagnostic methods on a large scale.

Haemozoin, produced when the malaria parasite digests haemoglobin, which is its primary nutrient source found as component of red blood cells, has been identified as a bio-marker for malaria diagnosis given its magnetic and birefringent properties [13–15]. As the parasite denatures haemoglobin, it creates haem, an iron-containing compound that is toxic to the parasite. To overcome this, the parasite converts haem into an insoluble crystalline form called haemozoin. In the Gazelle device, magnets repeatedly align haemozoin perpendicular to a strong magnetic field that is not retained when the field is removed. Haemozoin particles are birefingent and thereby the amount of polarized light that is transmitted through the sample is reduced proportional to the concentration of haemozoin in the sample [13, 16]. Based on this principle, a haemozoin-based, battery operated, malaria diagnostic device (Gazelle) was also evaluated for malaria diagnosis by the AMC.

This study aimed to compare a new haemozoin-detecting device, the Gazelle, with microscopy, Carestart™ RDT and nested PCR for screening and diagnosis of malaria in people suspected of having malaria over a period of six months.

## Methods

### Study setting and participants

This study was carried out between October 2020 and March 2021 at the Central Laboratory of the AMC Headquarters, Colombo, Sri Lanka. Two groups of individuals were screened for malaria (a) the first group comprised individuals referred to the AMC Headquarters (AMC HQ) Central Laboratory for confirmation of malaria based on clinical suspicion of disease. The reasons for clinicians suspecting and referring for confirmation of malaria included (i) travel history to a malaria endemic country, (ii) prolonged fever of unknown origin and, (iii) signs and symptoms suggestive of malaria, (b) the second group comprised individuals at high risk of malaria, namely security forces personnel returning from United Nations peacekeeping missions in Africa and individuals repatriated from African destinations who were screened by active case detection (ACD) by the AMC.

Proportion of agreement between the screening test and the gold standard was assumed as 50% to obtain the maximum sample size. Desired precision considered was 5%. α error was taken as 0.05. The calculated minimal sample size was 418 after allowing for a non-response rate of 10% [17].

### Sample collection

Two mL of intravenous blood was collected under aseptic conditions by trained nurses in to EDTA tubes after obtaining written informed consent from the participants to be included in the study. Blood samples of individuals suspected of having malaria were referred to the AMC by clinicians in state and government hospitals. In the case of high-risk individuals who had arrived from malaria endemic countries, the samples were collected within 24 h of arrival in Sri Lanka at the port of entry and transported to the AMC Headquarters.

### Laboratory diagnosis

The samples were screened for malaria by microscopy, Carestart™ RDT and Gazelle within 24 h of collection and PCR were carried out thereafter for confirmation of diagnosis. The assays were performed in a blinded manner using coded samples.

### Microscopy for malaria parasites

Thick and thin blood films were prepared from the collected blood samples. The slides were stained with Giemsa and screened for malaria parasites using microscopy, independently in a blinded manner by a PHLT, who is an expert microscopist and a parasitologist. A smear was interpreted as negative only after examination of 100 fields with an oil immersion lens at 1,000 magnification [18, 19]. If a smear was positive for malaria, at least 100 microscopic fields were screened to confirm the parasite species. Parasite density, expressed as the number of asexual parasites per microlitre of blood was calculate by dividing the number of asexual parasites for 200 WBCs counted and multiplying it by an assumed WBC density of 8000 cells/µL. In cases where there were fewer than 100 asexual parasites per 200 WBCs in smears, quantification was performed against at least 500 WBCs [18, 20]. Parasite densities were calculated by averaging the two counts obtained by the expert microscopist and parasitologist. Blood smears with non-concordant results (differences between the two microscopists in species diagnosis, or differences in parasite density of > 25%) were re-examined by a third, independent expert (WHO Certified Level 1 Microscopist), and parasite densities were calculated by averaging the two most concordant counts.

### Rapid Diagnostic Test (Carestart RDT)

*CareStart*™ Malaria Pf/PAN (HRP2/pLDH) Ag Combo Test, product G0131 (Access Bio Inc. USA) was used for malaria diagnosis. Performance of the tests and interpretation of the results were done according to the manufacturer’s instructions and the Standard Operating Procedures prepared by the AMC [21]. Briefly, five microlitre of blood was collected to the specimen transfer device supplied with the RDT kits. This was added to a sample well and then two drops of assay buffer solution was added to the buffer well. The test result was interpreted in 20 min. Individual band reactivity (HRP2 antigen for *P. falciparum* and pan-specific lactate dehydrogenase, pLDH) was recorded for all positive test results.

### Magneto-optical detection of malaria parasites by Gazelle

**Fig. 1:**
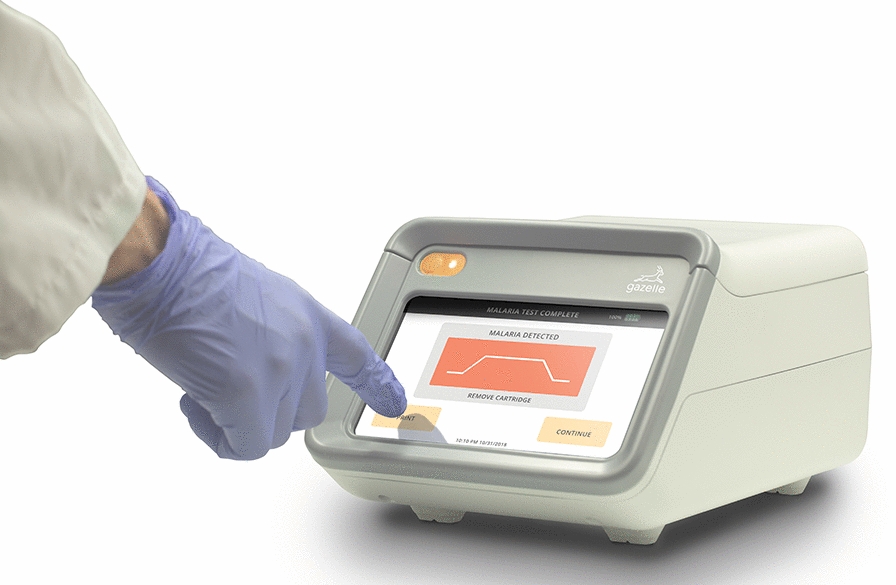
Table top Gazelle reader

**Fig. 2:**
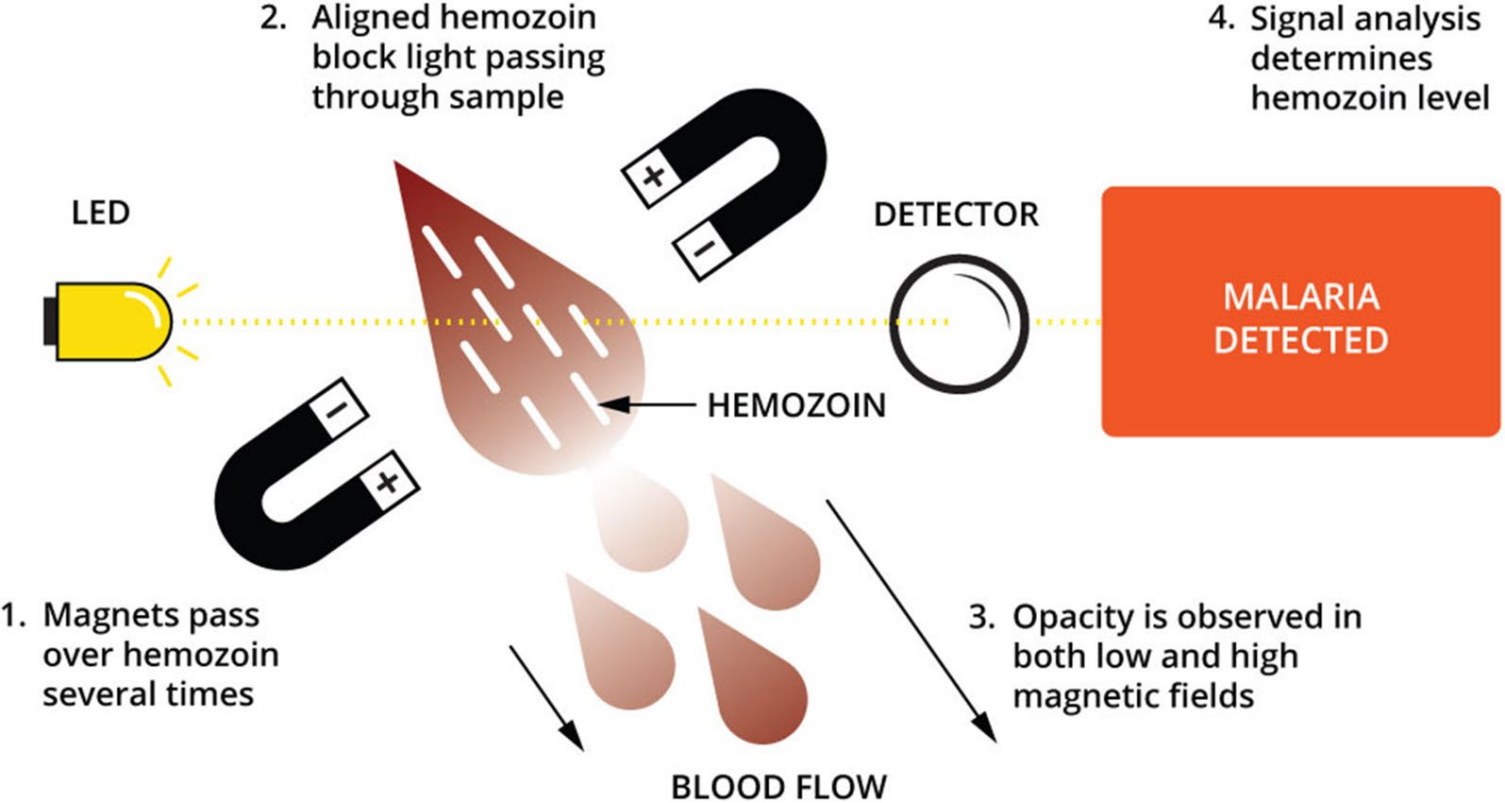
Principles of operation of Gazelle

A point-of-care portable magneto-optical device, Gazelle was also used for malaria diagnosis. Gazelle comprises a reader (small, tabletop) and single use disposable cartridges (Fig. 1). Prior to running samples, a positive and negative control was run on the reader to ensure appropriate functioning. As per manufacturer’s instructions [22, 23], 30 µL of the collected blood was taken with a pipette and inserted to the lower chamber of the Gazelle cartridge and thereafter the rest of the sample was stored for molecular diagnosis. 65 µL of the buffer was added to the Gazelle cartridge and the cartridge was inserted into the slot in the reader. The device was run and the results read within a minute and recorded. The individual performing Gazelle was blinded to the results of microscopy and Carestart RDT. Figure 2 depicts the principles of operation of Gazelle.

### Nucleic acid amplification assays (nPCR) for diagnosis

To confirm the presence or absence of parasites and parasite species, all samples were analysed by nested PCR assay at the AMC HQ Central Laboratory according to standardized protocols and published methods [24]. In brief, 200 µL of blood was used from whole blood samples. After washing 3 times, DNA was extracted using Qiagen DNA extraction kits according to manufacturer’s instructions. Extracted DNA was eluted in 200 µL Buffer AE. The purified DNA (~ 5 µL) was used as a template to detect malaria parasites using genus/species specific primers targeting 18S rRNA gene. Initially a *Plasmodium* genus specific nested PCR was carried out for all samples. Primers rPLU1/rPLU5 were used for the genus specific nest 1 reactions. The product of the first amplification reaction (1 µL) was used as the template for the genus specific second amplification reaction (nest 2) with the oligonucleotide primers rPLU 3 and rPLU 4. The base pair position of these primers with respect to the 18S rRNA A-type gene of *P. falciparum* (GenBank accession no. M19173) are for rPLU3 132–161 and for rPLU4 353–364 respectively. This gave a PCR product of approximately 235 bp. Whenever the genus-specific nest 2 PCR revealed positive results, the following species-specific nest 2 primers were used to determine the *Plasmodium* species: rFAL1/rFAL2 (*P. falciparum*), rVIV1/rVIV2 (*P. vivax*), rMAL1/rMAL2 (*P. malariae*), rOVA1/rPLU2 (*P. ovale*), and Pmk8/Pmkr9 (*P. knowlesi*). PCR products were electrophoresed for 30 min on a 2% (w/v) agarose gel at constant voltage of 120 V in 0.5 × Tris Borate EDTA buffer. The separated bands were visualized under an ENduro GDS UV trans illuminator (312 nm wave length) after staining for 15 min with ethidium bromide. Fragment sizes were estimated relative to the 100 bp ladder marker. A sample was considered as positive for *Plasmodium* if a DNA fragment of the expected size of 235 bp was visible. The products of the primary genus specific PCR reaction was used as the template for four separate species specific nested PCR assays.

### STARD adherence

STARD (Standards for Reporting of Diagnostic Accuracy Studies) shown in the Additional file 1: Fig. S1 details the study test results, including an analysis of accuracy compared with reference standards for microscopy and PCR (true and false positives and negatives), and comparison of results with RDT as a comparator (concordance and discordance between Gazelle™ and RDT results). The STARD checklist is shown in the Additional file 2: Table S1.

### Data analysis

The clinical data and laboratory results were collected on record forms and later entered into a Microsoft Excel database. Data recorded on the Gazelle was transferred to the parent company after each batch of samples was run.

The performance of Gazelle was calculated and compared with microscopy, RDT and PCR (the reference standard) with 95% confidence intervals for the following values: sensitivity, specificity, negative likelihood reactions, odds ratio and positive and negative predictive values (PPV, NPV). The total number of samples is those samples on which all four diagnostic tests were successfully performed.

## Results

### Characteristics of the population

**Fig. 3:**
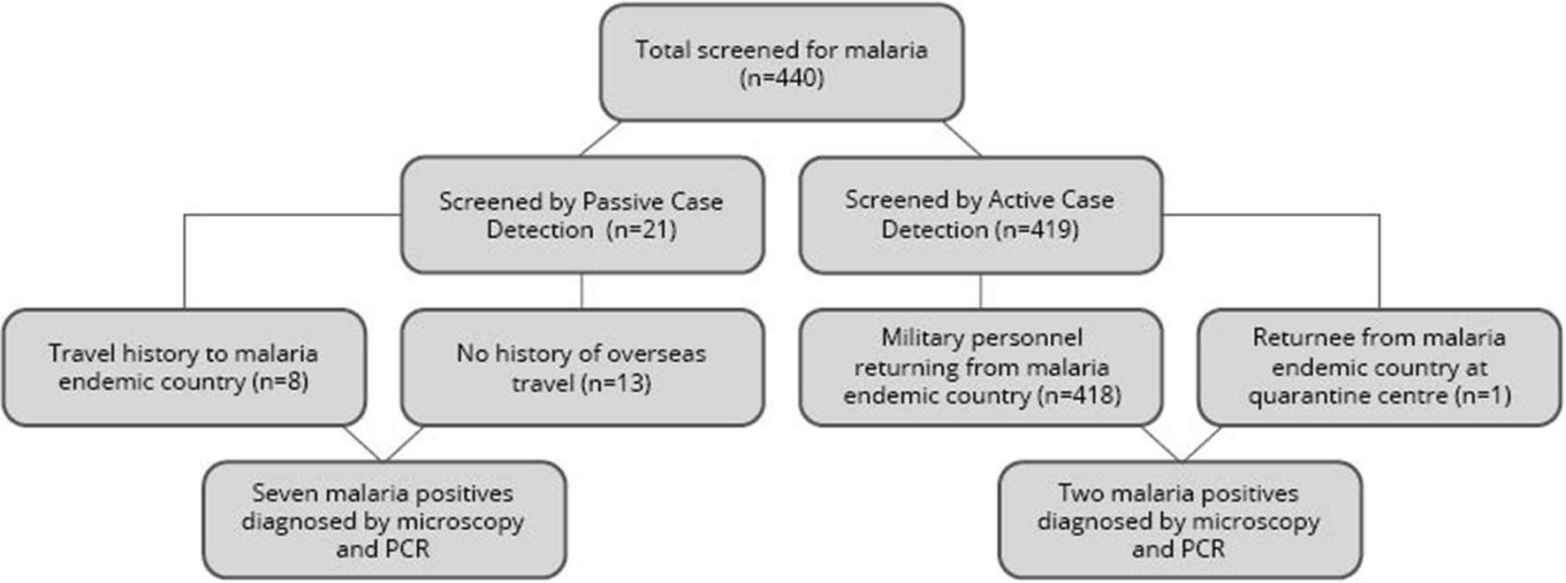
Flow chart depicting selection of study population. *Legend: 440 individuals were screened for malaria: 21 by passive case detection and 419 by active case detection*

440 individuals were screened for malaria over a six month period between October 2020 and March 2021. Of the 21 individuals that were screened by passive case detection eight gave a history of travel to a malaria endemic country (i.e. Uganda, Djibouti and Mozambique). The other 13 patients had not visited any overseas country within the past 1 year but were referred to exclude malaria in the differential diagnosis. The balance 419 were classified as a high-risk population for malaria and screened by ACD [7] (Fig. 3). 418 of these individuals were military personnel who had returned after a 12–14 month United Nations peacekeeping mission in African destinations (namely Central African Republic, South Sudan and Mali) and one individual was a returnee from Ethiopia who was at a quarantine centre and screened by ACD.

### Malaria detection

Nine malaria positive patients were diagnosed by microscopy (Table 1), which included seven individuals who were screened by PCD and had a travel history to a malaria endemic country, and two individuals diagnosed during screening by ACD. None of the individuals diagnosed with malaria had taken chemoprophylaxis during their overseas visit.

**Table 1:** Parasitological information of the individuals that were detected by different malaria diagnostic tests

Serial no	Species	Microscopy		Care-Start RDT		Gazelle	PCR
		Density at diagnosis	Stages	HRP2	LDH	Density range	Confirmed species
1	*P. vivax*	1904/μL	T/G	−	+	Positive	*P. vivax*
2	*P. falciparum*	339.8/μL	G	+	+	Positive	*P. falciparum*
3	*P. falciparum*	96/μL	R	+	−	Negative	*P. falciparum*
4	*P. falciparum*	1188/μL	R	+	−	Negative	*P. falciparum*
5	*P. falciparum*	9170/μL	R	+	+	Positive	*P. falciparum*
6	*P. malariae*	624/μL	T/S/G	−	+	Positive	*P. malariae*
7	*P. falciparum*	54698/μL	R	+	+	Positive	*P. falciparum*
8	*P. ovale*	901/μL	T/S/G	−	+	Positive	*P. ovale*
9	*P. ovale*	4815/μL	T/S/G	−	+	Positive	*P. ovale*

*R* Ring stage, *T* Trophozoite stage, *S* Schizont stage, *G* Gametocyte stage of malaria parasites, *HRP2* Histidine Rich Protein 2, *LDH* Lactate dehydrogenase enzyme

The nine malaria positive cases included five *P. falciparum*, two *P. ovale* and one each of *P. vivax* and *P. malariae* infections*.* The results of Carestart RDT (HRP2 band) and PCR were consistent with microscopy (Table 1). Gazelle however gave false negative results in two patients with *P. falciparum* infections.

### Comparison of diagnostic test results

The performance of the diagnostics methods tested, microscopy, RDT, PCR and Gazelle were compared. There was no significant difference between the number of samples tested with the positive results (n = 9), sensitivity (100%) and specificity (100%) between microscopy, RDT and PCR methods. The results of comparison of Gazelle with other methods are given in Table 2. As compared to microscopy, Gazelle had a sensitivity of 77.8% (100% for non-falciparum species, but missed two out of five *P. falciparum* cases). Specificity was 100% (Table 2). One missed *P. falciparum* case had a parasite density of 96/µl and the other 1188/μl (Table 1). The pLDH band of the Carestart RDT was also negative in these two cases (Table 1).

**Table 2:** Performance characteristics of diagnostic tests (n = 440)

	Microscopy (gold standard)/ RDT/PCR	
	Positive	Negative
Gazelle		
Positive	7	0
Negative	2	431
Sensitivity (95% Cl)	77.8% (40.0% to 97.2%)	
Specificity (95% Cl)	100.0% (99.2% to 100.0%)	
PPV(95% Cl)	100.0%	
NPV(95% Cl)	99.6% (98.5% to 99.9%)	
Accuracy (95% Cl)	99.6% (98.4% to 100.0%)	
Odds ratio	NA	
Positive likelihood ratio	NA	
Negative likelihood ratio	0.222	

Positive Likelihood Ratio = Sensitivity/(1-Specificity)

Negative Likelihood Ratio = (1- Sensitivity)/Specificity

DOR = (TP/FN)/(FP/TN) where DOR is Diagnostic Odds Ratio

The performance of the Gazelle was compared with the other diagnostic methods tested (i.e., microscopy, RDT and PCR). Similar results were obtained for all 3 comparisons. Specificity, sensitivity, PPV, NPV and accuracy (with 95% confidence intervals) for Gazelle as compared to microscopy, RDT and PCR are given in Table 2. Gazelle diagnosed only 7 malaria infections while all other methods detected 9 infections

## Discussion

This study aimed to determine the utility of the Gazelle as a rapid screening device for malaria infection, using typical populations where rapid screening would be an advantage. The low positive rate in this population limited the power to determine test accuracy. Compared to microscopy, RDT (HRP2) and PCR, Gazelle did not detect two *P. falciparum* malaria positives. However, during the study period, the Gazelle reader, which is a portable device, weighing less than 5 kg proved rapid and otherwise accurate in the rapid screening of blood samples. The benefits of using Gazelle in this study was identified as the need for low level of user training to perform the test, the ability to screen a large volume of samples in a short period of time and the rapidity of obtaining results.

The failure to detect the two ring stage infections of *P. falciparum* is a concern. Haemozoin has been demonstrated in early ring stages [25], but inability to detect the ring stages by Gazelle could reflect a lower haemozoin concentration in young parasitic stages, as identified elsewhere [26, 27]. Other haemozoin detection systems have claimed detection of *P. falciparum* at parasite densities below 40 parasites/uL at the ring stage [28, 29]. Gazelle’s sensitivity and specificity for diagnosis of low densities of *P. vivax* has also been shown to be similar to microscopy and better than RDTs [30].

Treatment in Sri Lanka is based on species identification and thus, until species identification is possible, Gazelle will not be able to be used as a sole test for diagnosis but it has the potential to be used as a screening test at special locations such as ports of arrival. The developer is developing a species-specific algorithm based on the variation in haemozoin crystal morphology between *Plasmodium* species [31]. Gazelle was accurately sensitive to diagnose both cases of *P. ovale* and *P. malariae* (noting the low numbers of these species in this study) which could prove useful due to the fact that Carestart RDT (pan specific-LDH) shows a lower sensitivity to diagnose both these species in Sri Lanka [10].

The blood samples collected for this study were transported to the AMC central laboratory where Gazelle was carried out. Should the test be carried out at a port of entry, it may prove to be cumbersome due to the requirement of pipettes to measure and insert the required blood volume and diluent into the Gazelle cartridge. This could be overcome by preparation of a one-step cartridge for Gazelle that could directly collect finger prick blood samples. This method is in development and should be available in the market in the near future.

In the absence of its ability to quantify and demonstrate the parasite species, Gazelle will be of advantage to be used in combination with HRP2 Carestart RDTs as a screening tool to screen large numbers of high-risk individuals arriving at ports of entry. Compared to the RDT which takes approximately 15–20 min, Gazelle can be performed in a very short time hence if the limitation regarding detection of *P. falciparum* can be addressed, it would be a better tool considering the very short time required for testing and giving the result.

## Conclusions

Gazelle, a haemozoin based malaria detecting device has potential for rapid throughput malaria screening in Sri Lanka due to its ease of use and availability of results within a short period of time. Sri Lanka is in the phase of prevention of re-establishment, where not only a limited number of malaria cases are diagnosed, but accurate diagnosis of each and every case is essential to prevent onward transmission. Thus, the technical challenges such as species identification, quantifying parasite density and ability to perform the test using finger prick blood should be addressed and overcome if it is to be used as a stand-alone screening device in a country such as Sri Lanka.

## Supplementary Information

**Additional file 1**: **Figure S1**. STARD Diagram.

**Additional file 2:** **Table S1**. STARD checklist for the reporting of studies of diagnostic accuracy. STARD Checklist.

## Data Availability

The datasets generated and/or analysed in this publication are not publicly available due to the fact that they belong to the Ministry of Health, Sri Lanka. Clarifications regarding data can be made through Dr. Prasad Ranaweera, Director of the Anti Malaria Campaign, Sri Lanka who is an author of this publication.

## Abbreviations

ACD: Active Case Detection
AMC: Anti Malaria Campaign
AMC HQ: Anti Malaria Campaign Headquarters
PCD: Passive Case Detection
PHLT: Public Health Laboratory Technician
POR: Prevention of re-establishment
RDT: Rapid Diagnostic Test
WHO: World Health Organization
