# Supplementary material for: Evaluation of a haemozoin-based rapid diagnostic test for diagnosis of imported malaria during the phase of prevention of reestablishment in Sri Lanka
Source: Malar J. 2022 Sep 10;21:263. doi: 10.1186/s12936-022-04283-7 (PMC9464370; doi:10.1186/s12936-022-04283-7)
Supplement: Supplementary file 1 — Additional file 1: Figure S1. STARD Diagram. [file 12936_2022_4283_MOESM1_ESM.pptx]

## Slide 1
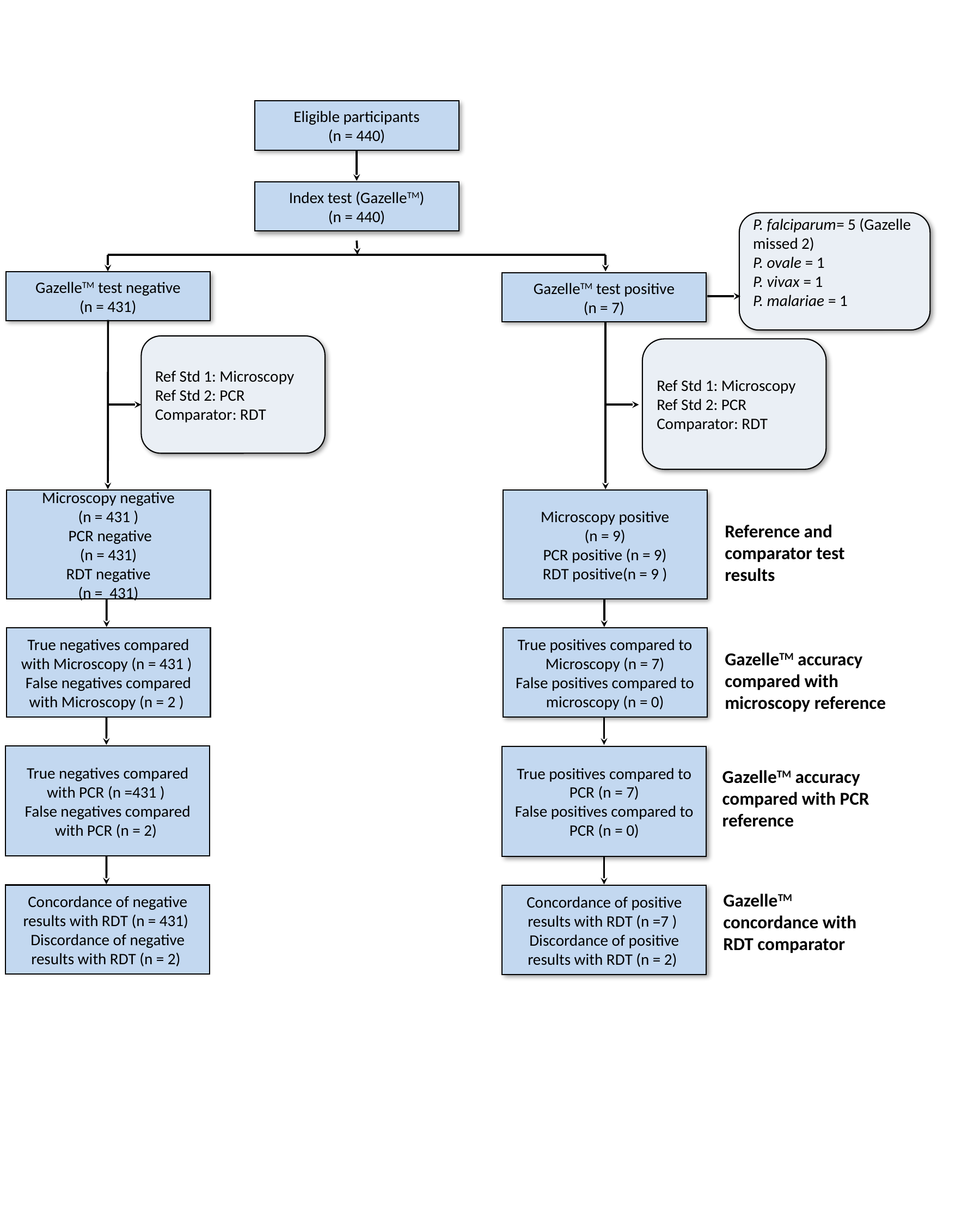

Eligible participants
(n = 440)
Index test (GazelleTM)
(n = 440)
P. falciparum= 5 (Gazelle missed 2)
P. ovale = 1
P. vivax = 1
P. malariae = 1
GazelleTM test negative
(n = 431)
GazelleTM test positive
(n = 7)
Ref Std 1: Microscopy
Ref Std 2: PCR
Comparator: RDT
Ref Std 1: Microscopy
Ref Std 2: PCR
Comparator: RDT
Microscopy negative
(n = 431 )
 PCR negative
(n = 431)
RDT negative
(n = 431)
True negatives compared with Microscopy (n = 431 )
False negatives compared with Microscopy (n = 2 )
True negatives compared with PCR (n =431 )
False negatives compared with PCR (n = 2)
Concordance of negative results with RDT (n = 431)
Discordance of negative results with RDT (n = 2)
Microscopy positive
(n = 9)
PCR positive (n = 9)
RDT positive(n = 9 )
Reference and comparator test results
True positives compared to
Microscopy (n = 7)
False positives compared to microscopy (n = 0)
GazelleTM accuracy compared with microscopy reference
True positives compared to
PCR (n = 7)
False positives compared to PCR (n = 0)
GazelleTM accuracy compared with PCR
reference
GazelleTM concordance with RDT comparator
Concordance of positive results with RDT (n =7 )
Discordance of positive results with RDT (n = 2)
